# Supplementary material for: Characterising phagocytes and measuring phagocytosis from live Galleria mellonella larvae
Source: Virulence. 2024 Feb 15;15(1):2313413. doi: 10.1080/21505594.2024.2313413 (PMC10877982; doi:10.1080/21505594.2024.2313413)
Supplement: Supplemental Material [file KVIR_A_2313413_SM2880.docx]

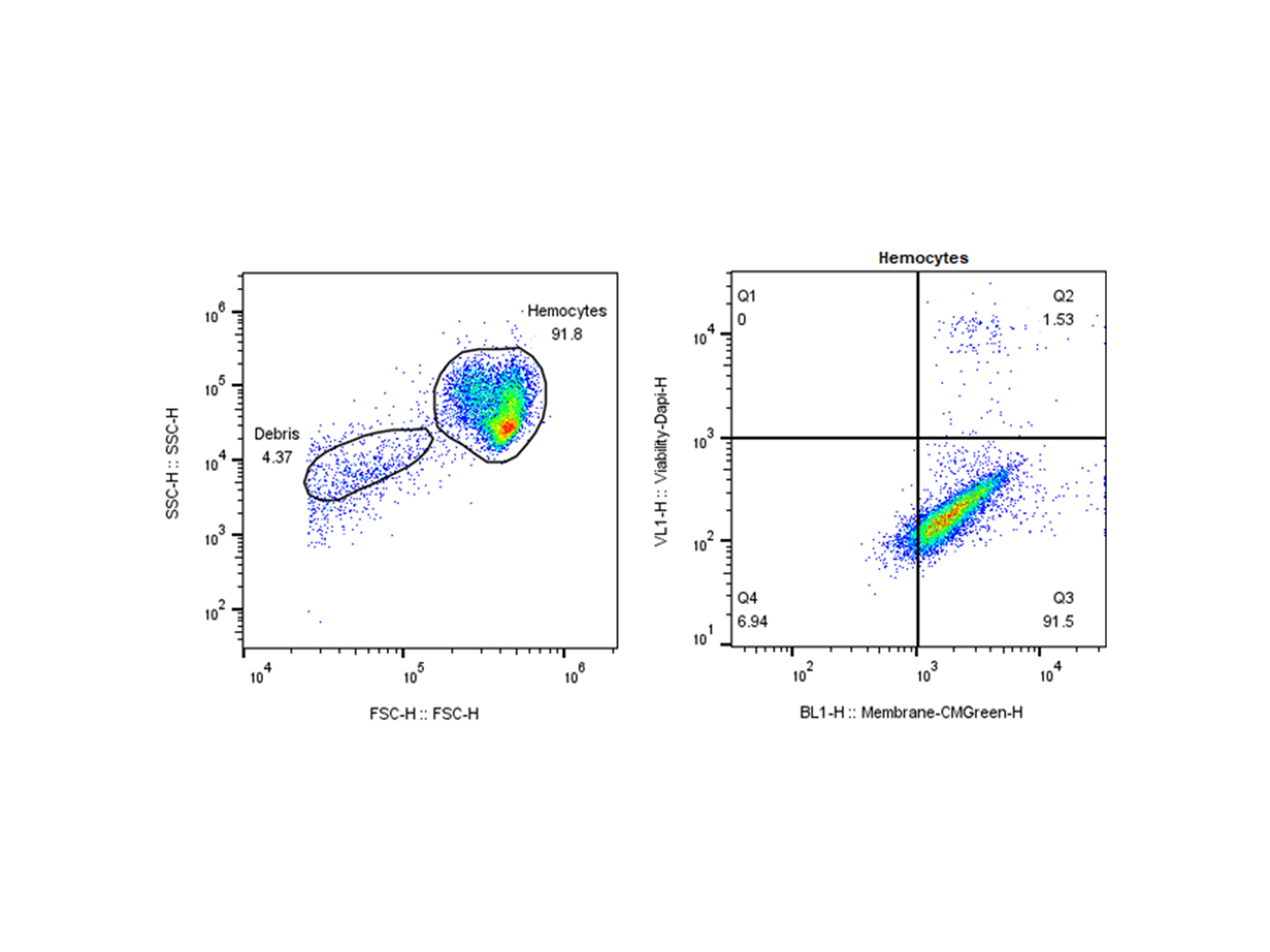


**Supplementary Figure S1**: A) FSC-H/SSC-H scatter plot showing gating placement for cells (hemocytes) within the hemolymph sample. B) Cell membrane dye (CMG-H) vs viability (Dapi-H) scatter signal of events within the hemocyte gate shown in A. Events are predominantly live cells (Q3 - 91.5%).
